# 多发性骨髓瘤伴胰腺、胃、皮肤多发髓外病变一例

**DOI:** 10.3760/cma.j.issn.0253-2727.2021.05.017

**Published:** 2021-05

**Authors:** 涛 王, 骥 谢, 飞雪 宋

**Affiliations:** 兰州大学第二医院肿瘤内科 730000 Department of Oncology, The Second Hospital of Lanzhou University, Lanzhou 730000, China

患者，女，50岁，因“全身骨痛两年余，加重两个月伴不能行走一周”入院。入院后行相关检查，CT及骨扫描提示：全身中轴骨及肋骨多发骨质破坏，颈、胸、全腹CT，头颅MRI未发现其他占位性病变，无皮肤、黏膜黄染，无皮疹、皮肤结节等。骨髓穿刺及活检示：骨髓中单克隆浆细胞占65％，免疫组化：CD38（+）、CD138（+）、κ轻链（+）。血清蛋白电泳：出现M条带，IgA 36 g/L、β球蛋白57.7％（参考值8.0％～13.0％）。尿本周蛋白2.8 g/24 h，分型：IgA κ型。血β_2_-微球蛋白4700 µg/L；血钙5.6 mmol/L；血生化常规示：球蛋白72.6 g/L（参考值20～40 g/L）。诊断为多发性骨髓瘤，Durie-Salmon分期ⅢA期，ISS分期Ⅱ期。

予BDT（硼替佐米+地塞米松+沙利度胺）方案化疗2个周期后生化学评估完全缓解，骨髓穿刺示：骨髓浆细胞3％；CT及MRI示：全身多发溶骨性骨质破坏区较前低密度区略缩小，边缘硬化，继续予BDT方案化疗4个周期，期间评估肿瘤稳定，患者临床症状缓解，活动自如，但未进一步巩固治疗。两个月后患者入院复查，IgA 7.11 g/L（参考值0.7～3.3 g/L），κ轻链（+），尿本周蛋白1.3 g/24 h，血β_2_-微球蛋白3800 µg/L；骨髓穿刺：骨髓浆细胞5％；影像学表现同前，评估生化学进展，建议患者继续维持治疗。遂再次予BDT方案维持治疗两个周期，两周期后评估生化学、影像学稳定，骨髓穿刺浆细胞3％，建议患者继续巩固治疗，患者及家属要求动态观察、随访。患者未定期复查。5个月后患者出现大小便困难，遂入院复查，实验室检查：IgA 11.01 g/L，κ轻链（+），尿本周蛋白1.6 g/24 h，血β_2_-微球蛋白4200 µg/L；骨髓穿刺：骨髓浆细胞5％；胸腹部CT示：全身多发溶骨性骨质破坏，多个胸腰椎压缩性改变，符合多发性骨髓瘤，骶骨右侧前方新出现软组织肿块；行MRI示：L1、L2椎体压缩性改变，S2-S5水平骶骨前方、L4-S5水平背部及骶管内多发异常软组织信号影，评估病情进展，遂行局部放疗36 Gy/12f，同时予口服来那度胺25 mg，第1～21天。放疗后复查MRI示骶尾骨前方团块样软组织肿块完全消失，患者排便困难消失，予来那度胺+多柔比星脂质体+地塞米松方案化疗2个周期，复查IgA 4.3 g/L，κ轻链（+），尿本周蛋白0.8 g/24 h，血β_2_-微球蛋白3100 µg/L；骨髓穿刺：骨髓浆细胞2％，继续原方案化疗2个周期后评估生化学及影像学稳定。

两个月后患者出现全身皮肤黏膜黄染，入院行胸腹部CT示：胰腺头颈部多发软组织肿块影，压迫胆总管下端致胆道系统扩张，胃底部软组织肿块影，腹壁软组织肿块影；肝功能：总胆红素380.77 µmol/L，直接胆红素232.81 µmol/L，间接胆红素147.96 µmol/L；IgA 16.53 g/L，κ轻链6.76 g/L（参考值1.56～4.08 g/L），尿本周蛋白2.4 g/24 h，血β_2_-微球蛋白5800 µg/L，HGB 85 g/L，肾功能正常；骨髓穿刺：骨髓浆细胞15％，行PTCD术，超声引导下胰腺、腹壁肿块穿刺活检及胃镜下胃内肿物活检，均显示肿块组织由分化程度不同的浆细胞构成，免疫组化染色：CD138（+），CD38（+），κ（+），λ（−），CD43（+），CD20（+），CD3（−），各标本Ki-67阳性指数50％～80％，结合上述结果，考虑浆细胞瘤髓外浸润，遂行胰头颈病灶放射治疗36 Gy/18f，同时予来那度胺+环磷酰胺+地塞米松方案化疗1个周期，治疗后胰头颈部肿块完全消退，黄疸消失，拔除PTCD管，并予原方案化疗2个周期，末次化疗期间全身皮肤多发大小不等结节（从头皮至足背），考虑髓外浸润。患者及家属放弃治疗，随访1个月后患者死亡。

